# Dicoumarol sensitizes hepatocellular carcinoma cells to ferroptosis induced by imidazole ketone erastin

**DOI:** 10.3389/fimmu.2025.1531874

**Published:** 2025-02-11

**Authors:** Ziwei Yang, Tixin Han, Ruibin Yang, Yinuo Zhang, Yifei Qin, Jialu Hou, Fei Huo, Zhuan Feng, Yaxin Ding, Jiali Yang, Gang Zhou, Shijie Wang, Xiaohang Xie, Peng Lin, Zhi-Nan Chen, Jiao Wu

**Affiliations:** ^1^ Department of Cell Biology, National Translational Science Center for Molecular Medicine, Fourth Military Medical University, Xi'an, Shaanxi, China; ^2^ State Key Laboratory of New Targets Discovery and Drug Development for Major Diseases, Department of Cell Biology, Fourth Military Medical University, Xi'an, China; ^3^ Shaanxi Key Laboratory of Bio-electromagnetic Detection and Intelligent Sensing, Military Biomedical Engineering School, Fourth Military Medical University, Xi'an, China; ^4^ Institutes of Biomedicine and Department of Cell Biology, Jinan University, Guangzhou, China

**Keywords:** hepatocellular carcinoma, ferroptosis, NRF2, NQO1, SLC7A11, prognostic model

## Abstract

**Introduction:**

Ferroptosis, an iron-dependent form of regulated cell death, is characterized by the lethal accumulation of lipid peroxides on cellular membranes. It not only inhibits tumor growth but also enhances immunotherapy responses and overcomes drug resistance in cancer therapy. The inhibition of the cystine-glutamate antiporter, system Xc–, induces ferroptosis. Imidazole ketone erastin (IKE), an inhibitor of the system Xc– functional subunit solute carrier family 7 member 11 (SLC7A11), is an effective and metabolically stable inducer of ferroptosis with potential in vivo applications. However, tumor cells exhibited differential sensitivity to IKE-induced ferroptosis. The intrinsic factors determining sensitivity to IKE-induced ferroptosis remain to be explored to improve its efficacy.

**Methods:**

Bulk RNA-sequencing data from hepatocellular carcinoma (HCC) and normal liver tissues were collected from The Cancer Genome Atlas (TCGA) and the Genotype-Tissue Expression (GTEx) databases. Differentially expressed genes were identified and intersected with the ferroptosis-related genes (FRGs) listed in the FerrDb database, yielding the identification of 13 distinct FRGs.

**Results:**

A ferroptosis signature index model (Risk Score) was developed to predict HCC prognosis. And SLC7A11 and NAD(P)H quinone dehydrogenase 1 (NQO1) were identified as candidate FRGs indicating poor prognosis of HCC. Dicoumarol (DIC), an inhibitor of NQO1, was subsequently employed to assess its sensitizing effects on IKE in HCC treatment. In HCC cell lines and the subcutaneous xenograft model, the combined suppression of SLC7A11 and NQO1 significantly enhanced the inhibitory effect on tumor growth by inducing ferroptosis.

**Discussion:**

In conclusion, our findings demonstrate that DIC sensitized HCC cells to IKE-induced ferroptosis in HCC. Moreover, the identification of potential drugs that enhance the susceptibility of HCC cells to ferroptosis could provide novel therapeutic strategies for the treatment of HCC.

## 1 Introduction

Hepatocellular carcinoma (HCC), the most prevalent form of liver cancer, ranks as the fourth leading cause of cancer-related mortality globally. It is characterized by a heterogeneous prognosis due to varying tumor burdens and the severity of chronic liver disease (1). Typically, HCC originates in cirrhotic livers, often associated with chronic liver diseases such as chronic hepatitis B or C infection, alcohol-related liver disease, and metabolic dysfunction-associated steatotic liver disease (2). For early-stage HCC, surgical therapies such as resection and transplantation offer substantial survival benefits. For intermediate-stage HCC, intra-arterial therapies, including transarterial embolization (TAE), transarterial chemoembolization (TACE), and transarterial radioembolization (TARE), serve as first-line treatments or bridging therapies before transplantation. However, HCC is often detected at advanced stages. Although tyrosine kinase inhibitors (TKIs)-based systemic therapies are widely used in advanced HCC, their clinical benefit is limited due to drug resistance (3). Immune checkpoint inhibitors have been approved for advanced HCC treatment, but the strong immunosuppressive tumor microenvironment inhibits cytotoxic T lymphocyte infiltration, restricting the responsiveness to immune checkpoint blockade in a minority of patients. Investigating resistance mechanisms and identifying novel therapeutic targets is critical for enhancing HCC therapeutic efficacy (2).

Tumor cells can develop resistance to antitumor drugs by promoting cell survival pathways, preventing apoptosis, and facilitating epithelial-mesenchymal transition. Defects in apoptosis contribute to resistance against cancer treatments and play a role in tumorigenesis. In human hepatocarcinogenesis, the dysregulation of the balance between cellular proliferation and death is a pro-tumorigenic principle (4). Some HCC patients exhibit poor responses to systemic therapies due to acquired or intrinsic resistance to apoptosis (5, 6). As resistance to apoptosis is a hallmark of cancer cells, the induction of non-apoptotic regulated cell death, such as ferroptosis, pyroptosis, and necroptosis, is emerging as a novel approach for cancer treatment (7). These targeted therapies have shown significant potential in enhancing therapeutic efficacy by bypassing apoptosis resistance and exhibiting synergistic antitumor immune responses (8).

In recent years, ferroptosis has emerged as a significant area of research due to its role as a natural tumor-suppressive mechanism and its potential to enhance antitumor immunity (9). Cancer cells, which require elevated levels of iron for survival, are particularly susceptible to ferroptosis, and this susceptibility is closely linked to the progression, treatment response, and metastasis of various cancer types (10). Notably, mesenchymal and dedifferentiated cancer cells, which are often resistant to apoptosis and conventional therapies, demonstrate a remarkable vulnerability to ferroptosis. The induction of ferroptosis can also restore the sensitivity of drug-resistant cancer cells to standard treatments (11). However, some cancer cells mitigate their susceptibility to ferroptosis by downregulating the ferroptosis pathway, leading to resistance to anticancer therapies. Furthermore, ferroptosis has been implicated in numerous oncogenic pathways, suggesting its potential as a target for novel cancer therapeutics (12). Emerging evidence indicates that ferroptosis exerts its anti-tumor effects by interacting with various tumor suppressor genes, highlighting its role as a tumor suppressor mechanism (13–16). Ferroptosis interacts with the tumor microenvironment (TME) in complex ways, influencing the immune response within TME. It has been reported that ferroptotic cells release pro-inflammatory damage-associated molecular patterns (DAMPs), which can trigger the innate immune system (17). High mobility group box 1 (HMGB1), one of the best-characterized DAMPs involved in immunogenic cell death, triggers inflammation and immune responses during ferroptosis induced by RAS-selective lethal 3 (RSL3) and erastin *in vitro* (18) (19). The glutathione (GSH)/glutathione peroxidase 4 (GPX4) axis is known to control the activities of lysyl oxidase (LOX) and prostaglandin-endoperoxide synthase (PTGS) *via* the peroxide (17). The enzyme PTGS2 serves as an effective marker of ferroptosis (20). The sensitivity of immune cells to ferroptosis in TME varies significantly; thus, regulating ferroptosis sensitivity may aid in the discovery of novel therapeutic strategies to improve cancer treatment (12). Pro-ferroptosis systems, which produce lipid peroxides, and ferroptosis defense systems, which detoxify these peroxides, exist in a delicate balance (21). When pro-ferroptosis activities exceed ferroptosis defense systems, excessive accumulation of lipid peroxides on cellular membranes can damage membrane integrity, ultimately leading to ferroptosis (21, 22). Oncogenes could cause ferroptosis resistance in cancer cells by stimulating antioxidant defense systems that prevent lipid peroxidation or by suppressing cellular metabolic processes that promote lipid peroxidation, including the formation of labile iron pools, the composition of polyunsaturated fatty acids (PUFA)-containing phospholipids, and the synthesis of mitochondrial reactive oxygen species. Identifying reliable biomarkers that can amplify pro-ferroptotic effects or increase the susceptibility of HCC tumors to ferroptosis may significantly contribute to a more accurate prognosis of HCC. This advancement could lead to the formulation of more efficacious therapeutic strategies, thereby enhancing survival rates among HCC patients (23). For example, activation of Yes-associated protein (YAP) signaling can sensitize HCC to ferroptosis *via* arachidonate lipoxygenase 3 (ALOXE3)-mediated lipid peroxidation accumulation (24).

Enhancing sensitivity to ferroptosis is critical for the application of ferroptosis-based therapeutic strategies. Bioinformatic analysis plays a significant role in studying cancer and ferroptosis. This study aims to identify ferroptosis-related genes (FRGs) and associated pathways in tumors, elucidate the regulatory mechanisms of ferroptosis within these malignancies, and assess the therapeutic efficacy and safety profiles of drugs targeting ferroptosis. Multiomics has identified the correlation between intratumor steatosis and the exhausted tumor immune microenvironment in HCC (25). Previous research has shown that a novel, integrated cell death index model predicts the prognosis and responsiveness to immune checkpoint inhibitors in patients with oesophageal squamous cell carcinoma (26). In a study of non-small cell lung cancer, thorough multi-omics analysis clarified the biology of cancer resulting from genetic aberration (27). Proteogenomic profiling of small cell lung cancer has been instrumental in uncovering distinct biological mechanisms and identifying subtype-specific therapeutic strategies (28).

Further investigation on the effectiveness of imidazole ketone erastin (IKE) in other animal cancer models would be beneficial. In this study, we identified two ferroptosis suppressor genes, solute carrier family 7 member 11 (SLC7A11) and NAD(P)H quinone dehydrogenase 1 (NQO1), associated with the prognosis of HCC by bioinformatics analysis. The combined inhibition of SLC7A11 and NQO1 had a more significant suppressive effect on tumor growth in the subcutaneous HCC model. These findings may inform whether ferroptosis stimulation can yield favorable therapeutic outcomes in specific cancer cases.

## 2 Materials and methods

### 2.1 Cell culture

Hep 3B cell line was obtained from Suzhou Starfish Biotechnology Co., Ltd. (TCH-C195). HCCLM3 cells were obtained from Be Na Culture Library (BNCC102270). Huh-7 and Hep G2 liver cancer cell lines were sourced from the American Type Culture Collection (ATCC, United States). Mycoplasma contamination testing confirmed all cell lines to be negative. The cells were maintained in a humidified incubator at 37°C with 5% CO2 and cultured in Dulbecco's Modified Eagle's Medium (DMEM), supplemented with 10% Fetal Bovine Serum (FBS).

### 2.2 Data acquisition and preprocessing

RNA-sequencing (RNA-seq) data of 50 normal tissues and 371 HCC patients were downloaded from The Cancer Genome Atlas (TCGA) (https://www.cancer.gov/tcga/) database. RNA-seq data of 110 normal tissues from the Genotype-Tissue Expression (GTEx) (https://www.genome.gov/) database and 369 overall survival (OS) data of HCC patients were obtained from the University of California Santa Cruz (UCSC) (https://xenabrowser.net/) database.

### 2.3 Identification of differently expressed FRGs in HCC

Three bioinformatics methods—DESeq2, edgeR, and limma—were employed to identify differentially expressed genes in 371 HCC cases and 160 normal samples. A significance threshold of *p* < 0.05 and log_2_ fold change (FC) > 1 were utilized as cut-off criteria. Principal component analysis (PCA) and heatmap visualizations of differential gene expression between tumor and normal tissues were generated using the R package "tinyarray." Using differential expression analysis methods—DESeq2, edgeR, and limma—we identified genes that were significantly up-regulated and down-regulated. Subsequently, we utilized the R package "tinyarray" to determine the intersection of these gene sets. A Venn diagram, representing 356 up-regulated genes and 154 down-regulated genes, was constructed using the R package "tinyarray." The intersection of these 510 genes with FRGs (functionally relevant genes) from FerrDb (http://www.zhounan.org/ferrdb/current/operations/download.html) was considered, leading to the identification of 13 differentially expressed FRGs in HCC. Volcano plots and heatmaps of the 13 FRGs were created using "tinyarray."

### 2.4 Enrichment analysis of differently expressed FRGs

The Gene Ontology (GO) and Kyoto Encyclopedia of Genes and Genomes Pathway (KEGG) enrichment analysis of 13 differently expressed FRGs was performed using "clusterProfiler," "org.Hs.eg.db," "enrichplot," "ggplot2," and "GOplot" R packages. We performed Gene Set Enrichment Analysis (GSEA) to further understand 13 differently expressed FRGs. Furthermore, GSEA analysis was also performed on the signaling pathways that were enriched in the high-risk and low-risk groups in HCC. Significance was set at a p-value threshold of less than 0.05. A thousand permutation analyses were run in order to assess the significance levels. We visualized co-expression potential with the R package "corrplot" to assess co-expression and anti-correlation between 13 differently expressed FRGs.

### 2.5 Protein-protein interaction (PPI) network and identification of hub genes and key molecules

The PPI network was utilized to identify functional modules based on 13 differently expressed FRGs. This was accomplished using Cytoscape (version 3.8.1) and the Search Tool for the Retrieval of Interacting Genes (STRING) database (https://string-db.org). The Molecular Complex Detection (MCODE) algorithm within Cytoscape and the CytoHubba plugin were employed to identify hub genes or significant PPI network modules.

### 2.6 Identification of prognostic genes and establishment of a prognostic model

A risk model incorporating univariate, multivariate, and lasso regression analysis was developed using the "ggExtra" and "survival" R packages. The model was then applied to stratify the patients into high-risk and low-risk groups.

### 2.7 Verification of the prognostic model

The correlation between gene expression levels and overall survival (OS) in HCC patients was investigated using data from the TCGA, International Cancer Genome Consortium (ICGC), and Gene Expression Omnibus (GEO) databases (GSE14520). Furthermore, receiver operating characteristic (ROC) curves for the 1-, 2-, and 3-year survival periods were employed to establish the gene expression cutoff that distinguishes high-risk or low-risk patients using the "forestplot" package's functionality for generating forest plots.

### 2.8 Assessment of risk genes

A nomogram, developed from a multivariate regression analysis, served as a prognostic tool using the "survival," "rms," and "regplot" R packages. This nomogram enabled the calculation of individual probabilities for clinical events by integrating various prognostic and determinant variables, such as age, gender, stage, and Risk Score, thereby facilitating the prediction of diverse prognoses influenced by gene expression. The calibration curve illuminated the accuracy of the model's probability estimation. A scatter plot, a common graphical representation of the calibration curve, depicted the model's predicted probabilities or scores on the x-axis and the empirically observed event rates on the y-axis. The "Corrplot" R package was utilized to evaluate the correlation between the Risk Score and expression of specific genes, including E-twenty-six-specific sequence variant 4 (ETV4), kinesin family member 20A (KIF20A), cyclin-dependent kinase inhibitor A (CDKN2A), SLC7A11, and NQO1.

### 2.9 Differential expression of the hub gene in different stages and screening of target genes

After identifying the Hub gene, we utilized the Gene Set Cancer Analysis (GSCA) database to compile and illustrate the progression of mRNA expression levels between early- and late-stage cancers (http://bioinfo.life.hust.edu.cn/GSCA/#/). Employing Origin's box plot functionality, we delineated the correlation between clinical stages and gene expression levels. Through an integrative approach involving PPI network analysis, risk assessment modeling, and a thorough review of pertinent literature, we identified three pivotal target genes. The "survival" R package was utilized to find the association between prognosis and three target gene expressions. In order to ascertain how genes interact, the Gene Transcription Regulation Database (http://gtrd20-06.biouml.org/) was utilized to investigate the transcription factors' downstream regulation of genes.

### 2.10 Drug resistance analyses

Utilizing the CellMiner tools, we rapidly retrieved transcript data for a comprehensive set of 22,379 genes and 360 microRNAs. Additionally, the platform offered activity reports for 20,503 chemical compounds, including 102 medications approved by the U.S. Food and Drug Administration. By translating the differential expression levels into quantifiable patterns across the National Cancer Institute (NCI)-60, we improved data organization and enabled sophisticated cross-comparisons through the application of an innovative pioneering pattern match tool. Utilizing samples from the CellMiner database, we quantified the relationship between drug sensitivity profiles and gene expression patterns. The drug binding affinity, depicted by the Z score, was plotted on the y-axis, with the x-axis indicating the gene's relative expression levels. The violin plot of the Z score under high and low gene expression was shown.

### 2.11 Multiplex immunohistochemistry (mIHC)

The high-throughput tissue microarray was procured from Shaanxi Avila Biotechnology Co., Ltd. (Cat. No. DC-liv11047). Within the 96 cases of the tissue microarray, 10 cases were normal liver tissue, and 86 cases were cancer patients (71 patients with HCC, 15 with intrahepatic cholangiocarcinoma, and 1 with mixed carcinoma). There were two panels of five biomarkers examined in this study, including panel 1: SLC7A11 (ab307601, Abcam, 1:100), NQO1 (393700, Thermofisher, 1:100), and NF-E2 p45-related factor 2 (NRF2) (R1312-8, Huabio, 1:100). Another tissue came from a subcutaneous tumor. And panel 2: HMGB1 (ab79823, Abcam, 1:300), PTGS2 (12282s, CST, 1:500). Formalin-fixed and paraffin-embedded (FFPE) samples were cut from subcutaneous tumors, sections of 5 μm thickness. The slides were stained manually according to the instructions using the Opal seven-color IHC Kit (NEL861001KT, PerkinElmer). Stained slides were scanned by the Vectra (Vectra 3.0.5, PerkinElmer). Representative images were used for analysis by the inform software after scanning (inform 2.3.0, PerkinElmer).

### 2.12 Western blotting

Briefly, cells were scraped into ice-cold radioimmunoprecipitation assay (RIPA) buffer, supplemented with a 1% final concentration of the Halt protease and phosphatase inhibitor cocktail. Cell lysates were centrifuged at 14,000 rpm for 15 min at 4°C, and supernatants were collected. Protein concentrations were determined using the Bradford method. Cellular proteins were separated by 10% SDS-gel electrophoresis (SDS-PAGE), transferred onto polyvinylidene fluoride (PVDF) membranes (0.45 μM, IPVH00010, Millipore), and blocked with 5% skim milk at room temperature for 1 h. Membranes were then incubated with the following primary antibodies overnight at 4°C: anti-cystine glutamate reverse transporter (xCT) (ab307601, Abcam, 1:1000), anti-NQO1 (393700, Thermofisher, 1:500), anti-NRF2 (R1312-8, Huabio, 1:1000), and anti-β-actin (ab8226, Abcam, 1:3000). They were washed and incubated with horseradish peroxidase conjugated goat anti-mouse IgG (H+L) (31430, Thermofisher, 1:5000) and goat anti-rabbit IgG (H+L) (31460, Thermofisher, 1:5000).

### 2.13 Immunofluorescence staining

HCCLM3 cells were seeded in 6-well plates at a density of 4 × 10^5^ cells per well. Following treatment with IKE, DIC, or IKE + DIC, cells were subjected to immunofluorescence analysis. They were fixed with 4% paraformaldehyde for 15 minutes and then washed with PBS. Subsequently, cells were permeabilized with 0.2% Triton X-100 to facilitate antibody penetration. Non-specific binding was blocked using a blocking buffer consisting of 5% normal goat serum (NGS) in PBS with 0.1% Tween-20 for 1 hour at room temperature. Primary antibodies, diluted in PBS with 1% BSA and 0.1% Tween-20, were applied to cells at 4°C overnight. The antibodies used were anti-cystine glutamate reverse transporter (xCT) (ab307601, Abcam, 1:100) and anti-NQO1 (393700, Thermo Fisher Scientific, 1:100). After incubation, unbound primary antibodies were washed away with PBS. Fluorochrome-conjugated secondary antibodies, compatible with the primary host species, were then applied for 1 hour at room temperature in the dark. Following further washing, cell nuclei were counterstained with DAPI. Immunofluorescence was visualized using a confocal microscope with appropriate filters for the fluorochromes.

### 2.14 Measurement of cell death

Four HCC cell lines (Hep G2, Hep 3B, HCCLM3, and Huh-7) were seeded in 96-well plates (3 × 10^3^ cells/well). IKE (TS#2301, TargetingScience) was diluted to 8 μM, 4 μM, 2 μM, and 1 μM, respectively. Dicoumarol (DIC) (HY-N0645, MCE) was diluted to 4 μM. Ferrostatin-1 (HY-100579, MCE) was used as a specific ferroptosis inhibitor. Cells were collected for SYTOX Green (S7020, Invitrogen) and Hoechst33342 (C1029, Beyotime) staining for analyzing cell death by microscopy, and the number of dead cells and total cells was counted using ImageJ software.

### 2.15 *In vivo* xenograft mouse model

Male nude mice (7 weeks old) were injected with 3.0 × 10^6^ HCCLM3 suspension cells subcutaneously. Tumor size was measured by an electronic caliper every 2 days and calculated using the formula: tumor size (mm^3^) = 0.5 × length × width^2^. Mice were randomly separated into 4 groups, 8 mice per group. When the average tumor volume of each group of mice reached 100 mm^3^, drug intervention was started. One group of mice was injected with the solvent (5% DMSO + 5% Tween-80 + 40% PEG-300 + 50% ddH_2_O) as a control. Two groups of mice were injected with IKE or DIC, respectively. One group of mice was injected with IKE and DIC at the same time. All three drugs were injected intraperitoneally. IKE was injected continuously for two weeks, 100 μL (30 kg/ml) each time. DIC was injected every other day for two weeks, 100 μL (30 kg/ml) each time.

### 2.16 Statistical analysis

Statistical analyses were performed using R software (version 4.2.3) and GraphPad Prism 8 (GraphPad Software Inc., USA). Comparisons between two groups were made for normally distributed variables using an independent Student's t-test, while non-normally distributed variables were assessed using the Mann-Whitney U test (Wilcoxon rank-sum test). The experimental data are presented as the mean ± SEM. Descriptive statistics were calculated using GraphPad Prism. Statistical significance was defined as *p* < 0.05.

## 3 Results

### 3.1 Identification of 13 differently expressed FRGs in HCC

**Figure 1:**
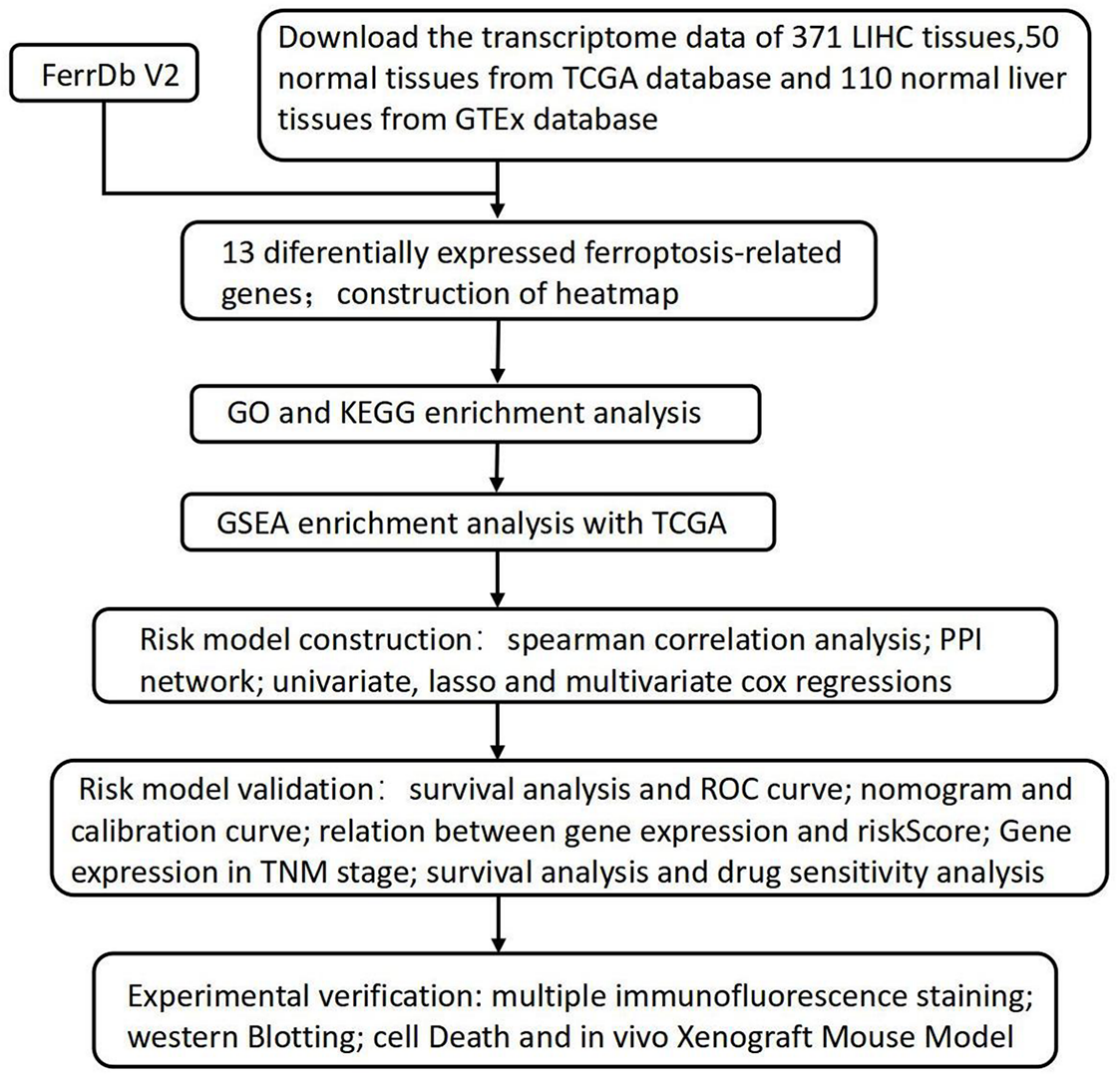
The flowchart of the research process.

**Figure 2:**
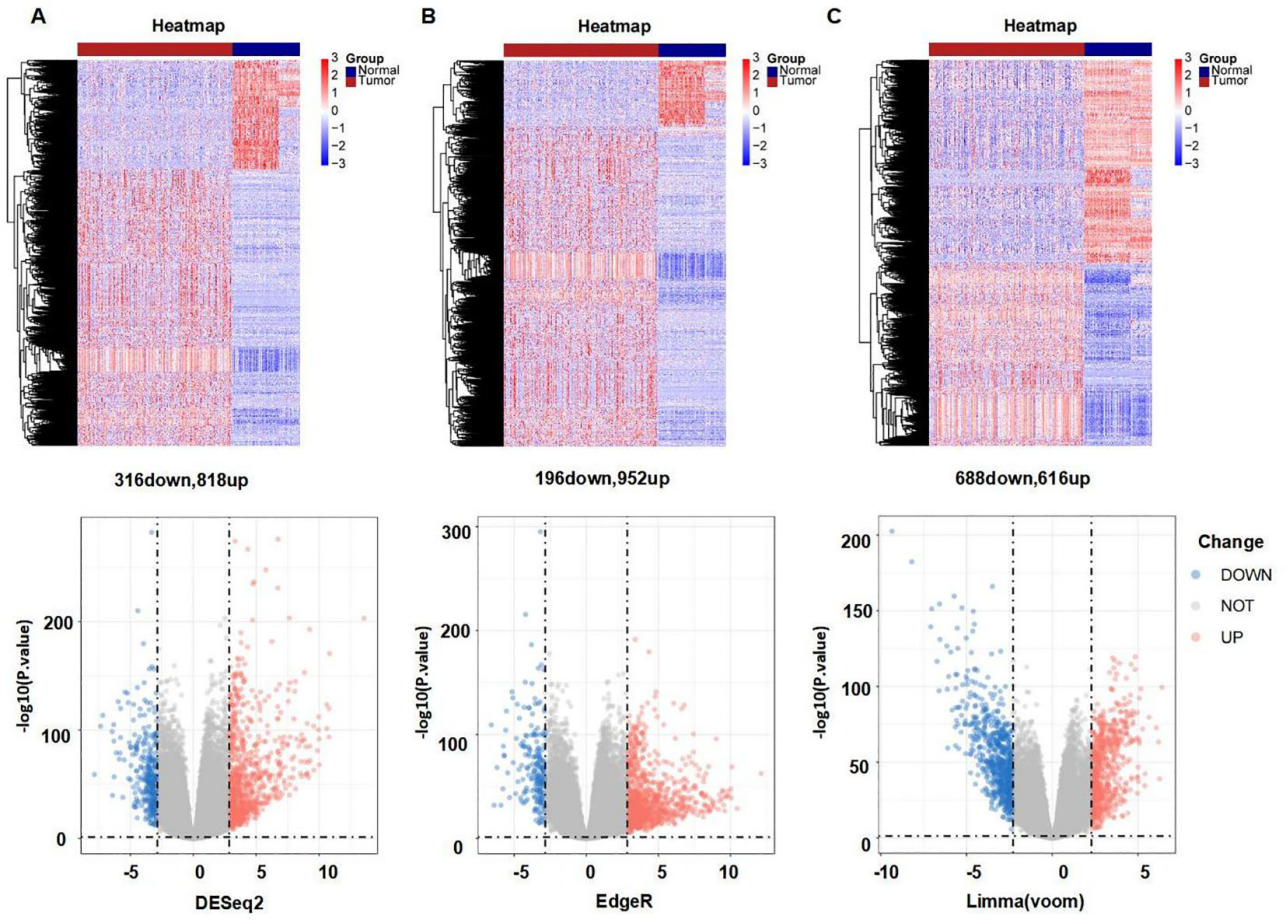
Differently expressed FRGs between HCC tissues and normal tissues analyzed with three data analysis methods. **(A)** Heatmap and volcano plot indicating FRGs using "DESeq2." **(B)** Heatmap and volcano plot indicating FRGs using "edgeR." **(C)** Heatmap and volcano plot indicating FRGs using the "limma" R package. Red indicating high expression, blue indicating low expression, "Normal" representing normal tissues, and "Tumor" representing tumor tissues. Volcano plots of differently expressed FRGs, with red indicating up-regulated, blue indicating down-regulated, "Up" representing up-regulated genes, "Down" representing down-regulated genes, and "NOT" representing neither up-regulated genes nor down-regulated genes.

**Figure 3:**
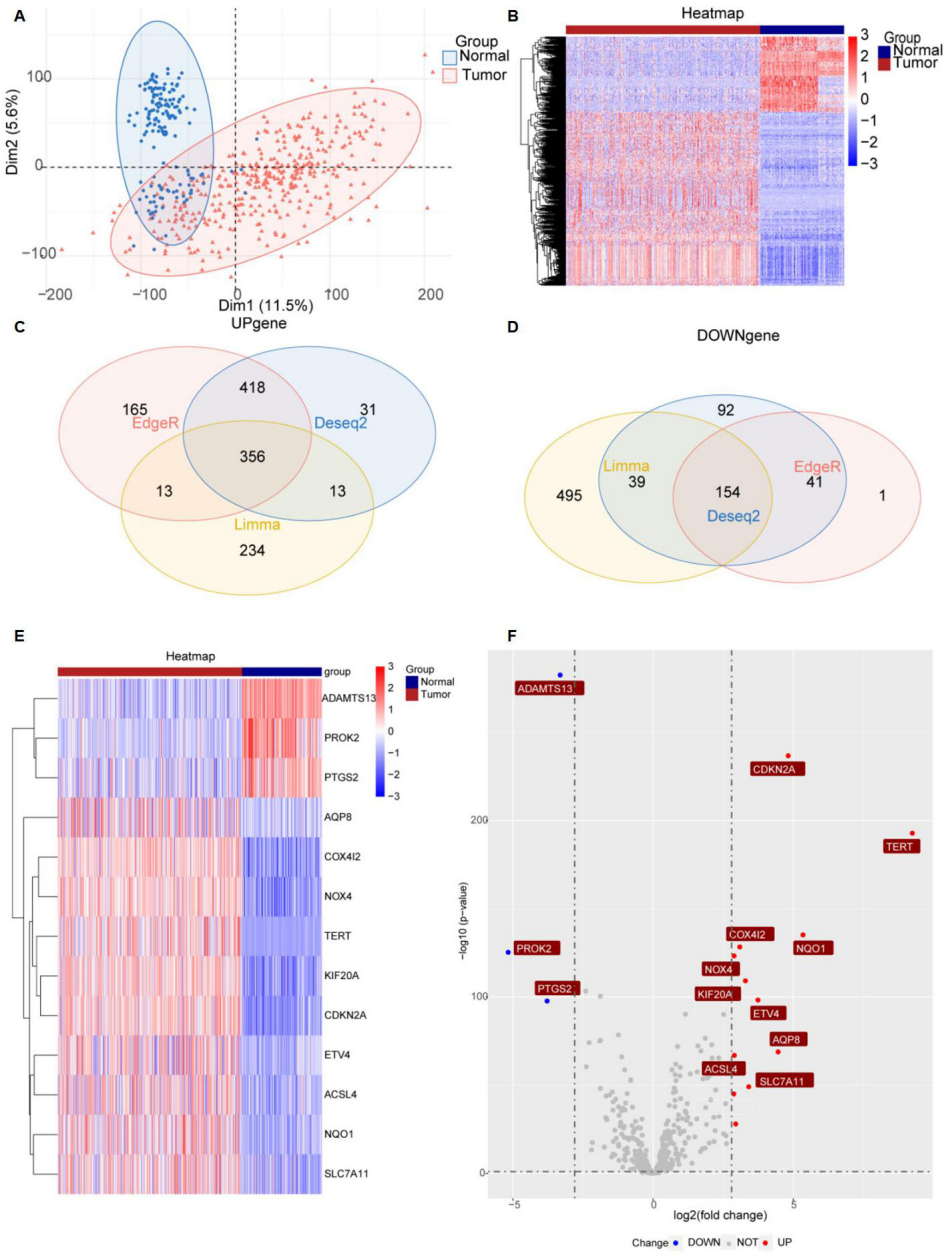
Significantly differently expressed FRGs are screened between HCC tissues and normal tissues. **(A)** PCA diagram of dimensionality reduction analysis, with red indicating tumor, blue indicating normal, "Normal" representing normal tissues, and "Tumor" representing tumor tissues. **(B)** Heatmap of differently expressed FRGs, with red indicating high expression, blue indicating low expression, "Normal" representing normal tissues, and "Tumor" representing tumor tissues. **(C, D)** Venn diagram showing the overlap of differentially expressed genes in the TCGA and GTEx databases. **(E, F)** Heatmap and volcano plots of 13 differently expressed FRGs, with red indicating high expression, blue indicating low expression, "Normal" representing normal tissues, and "Tumor" representing tumor tissues. "Up" representing up-regulated genes, "Down" representing down-regulated genes, and "NOT" representing neither up-regulated genes nor down-regulated genes.

**Figure 1** provides a comprehensive overview of the bioinformatics analysis workflow employed in this study. FRGs were extracted from the FerrDb V2 database. Liver cancer-related genes were identified in 110 normal tissues from GTEx and in 50 normal tissues and 371 HCC cases from the TCGA database. Three distinct bioinformatics methods were utilized to analyze differentially expressed genes in HCC, with results depicted in a heatmap and a volcano plot. Using the R package "DESeq2," we identified 316 down-regulated and 818 up-regulated differently expressed FRGs (**Figure 2A**). With the R package "edgeR," 196 down-regulated and 952 up-regulated genes with differential expression related to ferroptosis were found (**Figure 2B**). The "limma" package revealed 688 down-regulated and 616 up-regulated FRGs (**Figure 2C**). Principal component analysis (PCA), a dimensionality reduction technique, distinctly showed the distribution patterns between HCC patients and healthy individuals (**Figure 3A**). Heatmaps were used to illustrate the gene expression profiles of HCC patients and healthy individuals (**Figure 3B**). A Venn diagram highlighted the overlap and uniqueness of up-regulated and down-regulated, revealing differential expression patterns. By integrating results from DESeq2, edgeR, and limma, we identified a common set of 356 up-regulated and 154 down-regulated genes in HCC, indicating significant alterations in gene expression (**Figures 3C, D**). The intersection of these genes with FRGs yielded 13 differentially expressed FRGs in HCC. These genes were visualized using a heatmap and a volcano plot to show their expression patterns and the significance of their expression changes (**Figures 3E, F**). The heatmap indicated that aquaporin-8 (AQP8), cytochrome c oxidase subunit 4 isoform 2 (COX4I2), NADPH oxidase 4 (NOX4), telomerase reverse transcriptase (TERT), KIF20A, CDKN2A, ETV4, acyl-CoA synthetase long-chain family member 4 (ACSL4), NQO1, and SLC7A11 were down-regulated in tumors relative to normal tissues, whereas a disintegrin and metalloproteinase with thrombospondin type 1 motif, 13 (ADAMTS13), prokineticin 2 (PROK2), and PTGS2 were up-regulated.

### 3.2 Identification of gene correlation through functional enrichment analysis, gene set enrichment analysis, and gene correlation analysis

**Figure 4:**
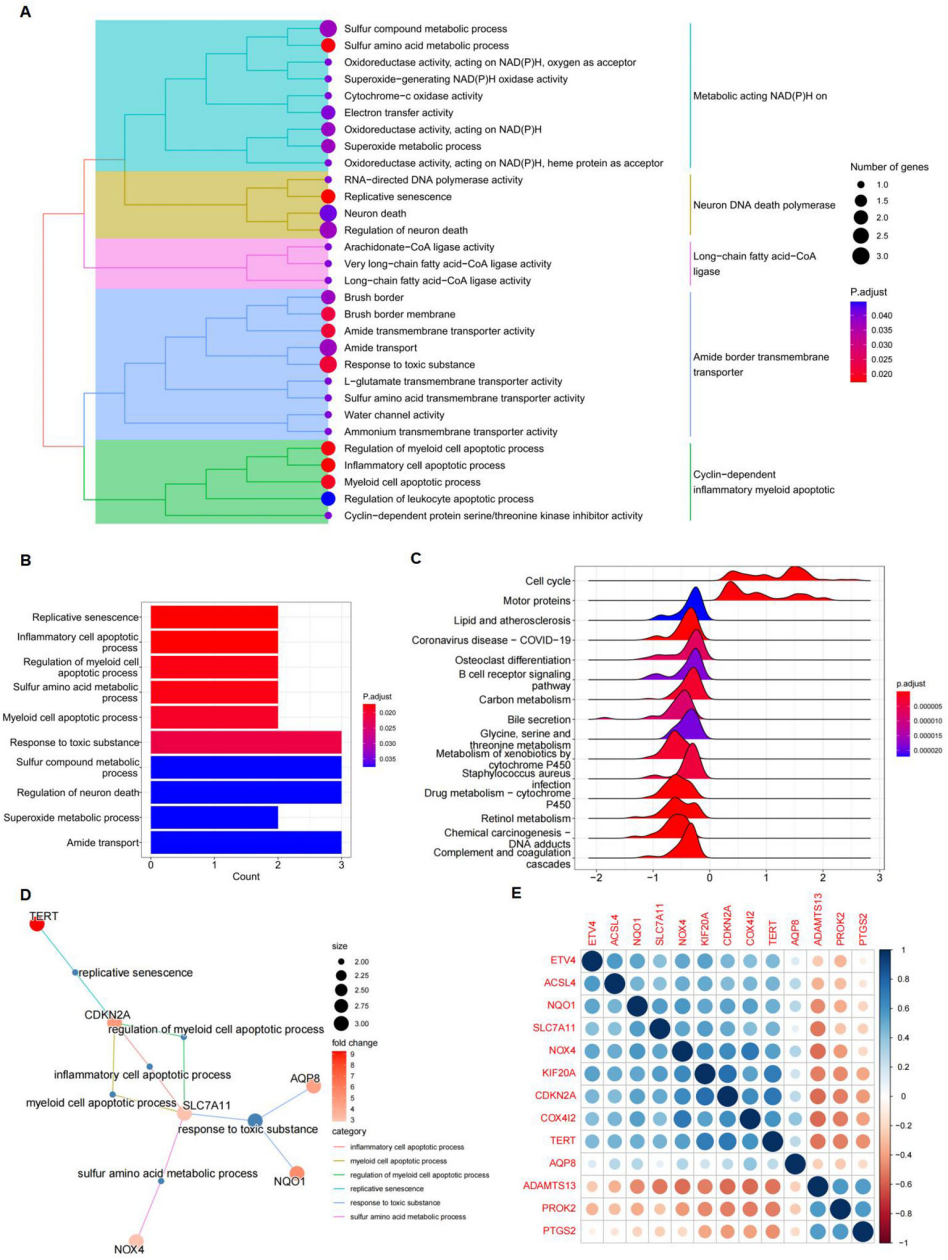
Gene function analysis of significantly differently expressed FRGs. **(A)** A treeplot of GO and KEGG enrichment analysis. **(B)** Barplot of GO and KEGG enrichment analysis. **(C)** Ridge diagram of 13 differently expressed FRGs. **(D)** Cnetplot of GO and KEGG enrichment analysis. **(E)** A positive correlation between NQO1 and SLC7A11. Gene-by-gene correlation matrix visualizing the pairwise Pearson correlation coefficients in bulk RNA-seq TCGA data from patients with HCC (n = 371). Genes are favorably connected if their respective circles are near blue; on the other hand, genes are negatively correlated if their respective circles are near red.

GO and KEGG pathway enrichment analyses were performed to investigate the functions of the differentially expressed FRGs. Treemap visualizations demonstrated that these FRGs were predominantly linked to replicative senescence, inflammatory cell apoptosis, sulfur amino acid metabolism, and regulation of myeloid cell apoptosis (**Figure 4A**). The data were graphically represented using a treeplot for hierarchical detail and a barplot for an overview of enrichment scores (**Figures 4A, B**). GSEA was applied to identify the most relevant phenotypic pathways among the differentially expressed FRGs. The ridge diagram indicated significant enrichment of these genes in various biological pathways, including the cell cycle, complement and coagulation cascades, drug metabolism (cytochrome P450), retinol metabolism, coronavirus disease (COVID-19), carbon metabolism, and retinol metabolism (**Figure 4C**). A cnetplot visually depicted significant gene-gene interactions, particularly the strong association between SLC7A11 and CDKN2A (**Figure 4D**). In the TCGA HCC patient cohort RNA-seq data, which comprised 371 subjects, pairwise Pearson correlation coefficients were calculated to evaluate gene-gene relationships and were visualized in a matrix format for gene-by-gene comparison. A subsequent correlation analysis focused on the 13 differentially expressed FRGs and confirmed the correlation between ferroptosis and these genes (**Figure 4E**).

### 3.3 Establishment of a prognostic model and identification of prognostic FRGs

**Figure 5:**
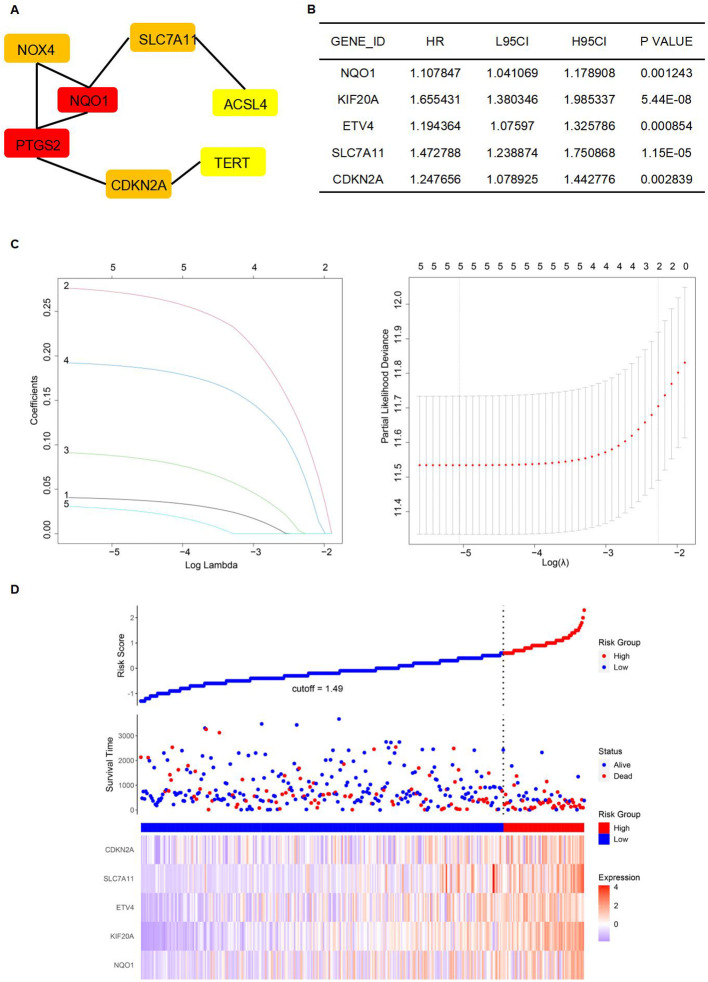
Establishment of a prognostic model. **(A)** The establishment of the PPI network. **(B)** Genes were significantly expressed in the univariate Cox regression model. **(C)** Lasso regression of 5 genes in **(B)**. **(D)** High-risk and low-risk groups according to Risk Score.

A protein-protein interaction (PPI) network was constructed to identify key genes and visualize their interactions. The string interaction network of genes with varying expression levels was investigated using Cytoscape. Utilizing the MCODE plugin within Cytoscape, we identified highly interconnected subnetworks, suggesting the presence of functionally related genes. Specifically, we selected seven genes to construct a PPI network relevant to ferroptosis: SLC7A11, NQO1, TERT, NOX4, ACSL4, PTGS2, and CDKN2A (**Figure 5A**). Among these, SLC7A11, NQO1, and TERT could inhibit ferroptosis (29–31). To identify FRGs whose expression levels correlate with the overall survival (OS) of HCC patients, we conducted a univariate Cox proportional hazards regression analysis. This approach allowed us to assess the association between gene expression and survival outcomes, applying a threshold p-value to refine potential prognostic FRGs. Notably, NQO1, KIF20A, ETV4, SLC7A11, and CDKN2A exhibited significant differential expression (**Figure 5B**). Lasso regression, incorporating a regularization parameter λ, was then applied to identify the optimal predictive model, reducing regression coefficients β and eliminating variables with minimal impact. Lasso regression mitigates multicollinearity and overfitting by penalizing regression coefficients. Cross-validation indicated improved predictive accuracy as the deviation from the partial likelihood ordinate decreased. NQO1, KIF20A, ETV4, SLC7A11, and CDKN2A were identified as having the best fit within the model (**Figures 5B, C**). Furthermore, the multivariate Cox regression analysis identified NQO1, KIF20A, ETV4, SLC7A11, and CDKN2A as statistically significant predictors of increased mortality risk in HCC patients, suggesting their potential as independent risk factors and therapeutic targets. Subsequently, a stepwise multivariate Cox regression analysis was used to develop prognostic indicators, identifying genes with significant prognostic value. The prognostic Risk Score for each patient was calculated as the weighted sum of gene expression levels, each multiplied by its corresponding regression coefficient from the multivariate Cox regression analysis: Risk Score = 0.042*NQO1+0.281*KIF20A+0.095*ETV4+0.195*SLC7A11+0.034*CDKN2A (**Figure 5D**).

### 3.4 Assessment and verification of the prognostic model

**Figure 6:**
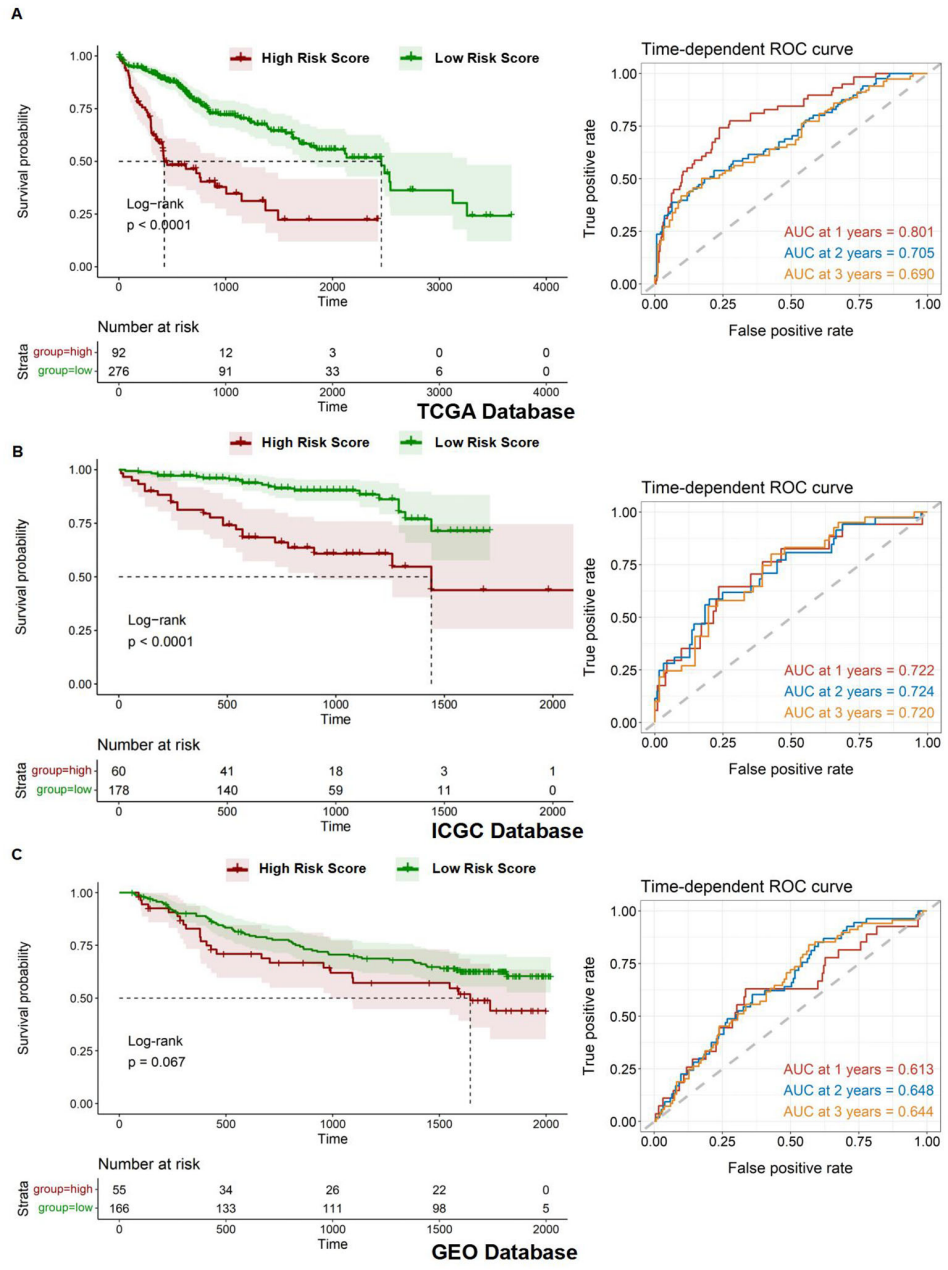
Assessment of prognostic model. **(A-C)** Kaplan-Meier overall survival (OS) curves for patients with high and low Risk Scores and time-dependent ROC curves in the TCGA, ICGC, and GEO databases.

After establishing the prognostic risk model, we validated its performance using three independent datasets: TCGA, ICGC, and GEO. The Risk Score for each patient was calculated using the multivariate Cox regression method, which was followed by survival analyses. Patients were subsequently stratified into high- and low-risk groups based on their Risk Score. To evaluate the predictive accuracy of the model, we constructed a ROC curve. The area under the curve (AUC) value represents the model's ability to distinguish between high-risk and low-risk groups within each corresponding database. It is observed that the AUC varies under different survival times for patients. The survival time with the highest AUC value can be selected to assess patient risk, indicating the optimal discrimination between the high- and low-risk groups. The AUC values for the 1-year, 2-year, and 3-year survival predictions for HCC patients were 0.801, 0.705, and 0.690, respectively, demonstrating significant model performance with a p-value of less than 0.0001 for the Kaplan-Meier (Km) survival analysis within the TCGA database (**Figure 6A**). Similarly, the AUC values for the HCC patients' 1-year, 2-year, and 3-year survival predictions were 0.722, 0.724, and 0.720, respectively, confirming the model's robustness with a p-value of less than 0.0001 for the Km survival analysis under the ICGC database (**Figure 6B**). Lastly, the AUC values for the 1-year, 2-year, and 3-year survival predictions were 0.613, 0.648, and 0.644, respectively, with a p-value of less than 0.067 for the Km survival analysis in the GEO database (**Figure 6C**).

### 3.5 Assessment of the risk genes by Risk Score

**Figure 7:**
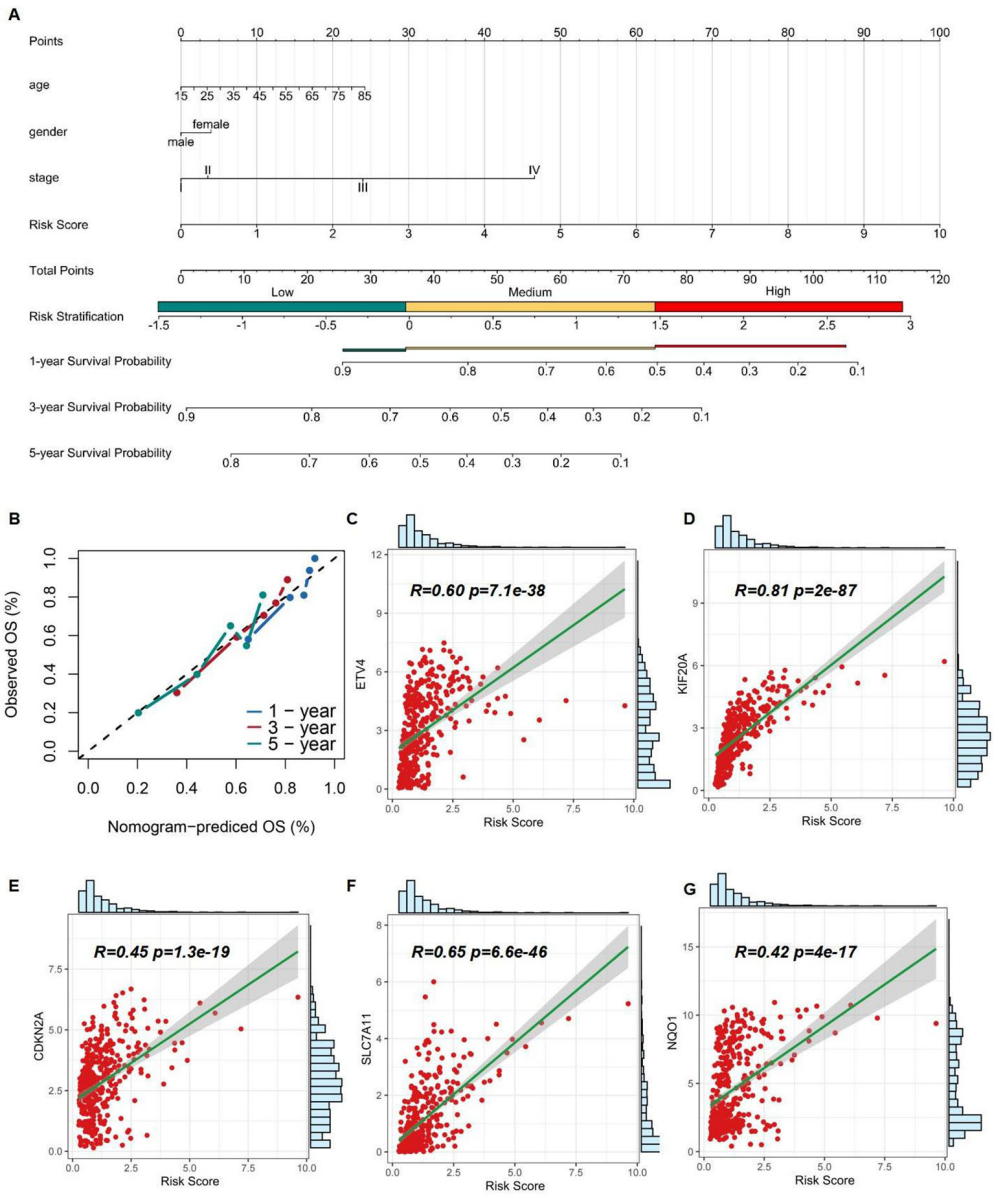
Assessment of 5 risk genes. **(A)** A nomogram based on multivariate regression. **(B)** Calibration curve of the risk model. **(C-G)** Correlation of Risk Score and expression of ETV4, KIF20A, CDKN2A, SLC7A11, and NQO1.

The nomogram was designed into three main components (1): Predictive model variables, including age, gender, and stage, were clearly labeled and accompanied by a visual depiction of the Risk Score. Each variable was allocated a line segment on the nomogram, calibrated with a scale that matched its value range. The length of these line segments represented the relative impact of each variable on the final outcome (2). The total score, labeled as ''Total Points'' on the nomogram, was determined by aggregating the individual scores from the variable values. Each variable's contribution was quantified by a score, graphically displayed as a point along the corresponding line segment (3). The nomogram detailed survival probabilities, noting that for the ''High Risk Stratification'' group, the 1-year survival rate was the primary endpoint, with the 5-year survival rate being less probable. Conversely, the ''Low Risk Stratification'' group was characterized by significant 3- and 5-year survival rates as the main outcomes. (**Figure 7A**). Ideally, the model's scatter points should align with a 45-degree line, indicating a close match between predicted and observed outcomes. Our analysis showed increasing predictive accuracy for 5-year, 1-year, and 3-year survival probabilities (**Figure 7B**). Using the TCGA database, we developed a nomogram model to calculate the Risk Score for HCC patients and correlated it with the expression levels of five pre-screened genes. Each gene exhibited a significant correlation: ETV4 (correlation coefficient R = 0.60, p-value = 7.1e-38), KIF20A (R = 0.81, p-value = 2e-87), CDKN2A (R = 0.45, p-value = 1.3e-19), SLC7A11 (R = 0.65, p-value = 6.6e-46), and NQO1 (R = 0.42, p-value = 4e-17), highlighting their potential as prognostic biomarkers (**Figures 7C–G**).

### 3.6 Higher expression of NQO1 and SLC7A11 is correlated with a poorer prognosis in HCC

**Figure 8:**
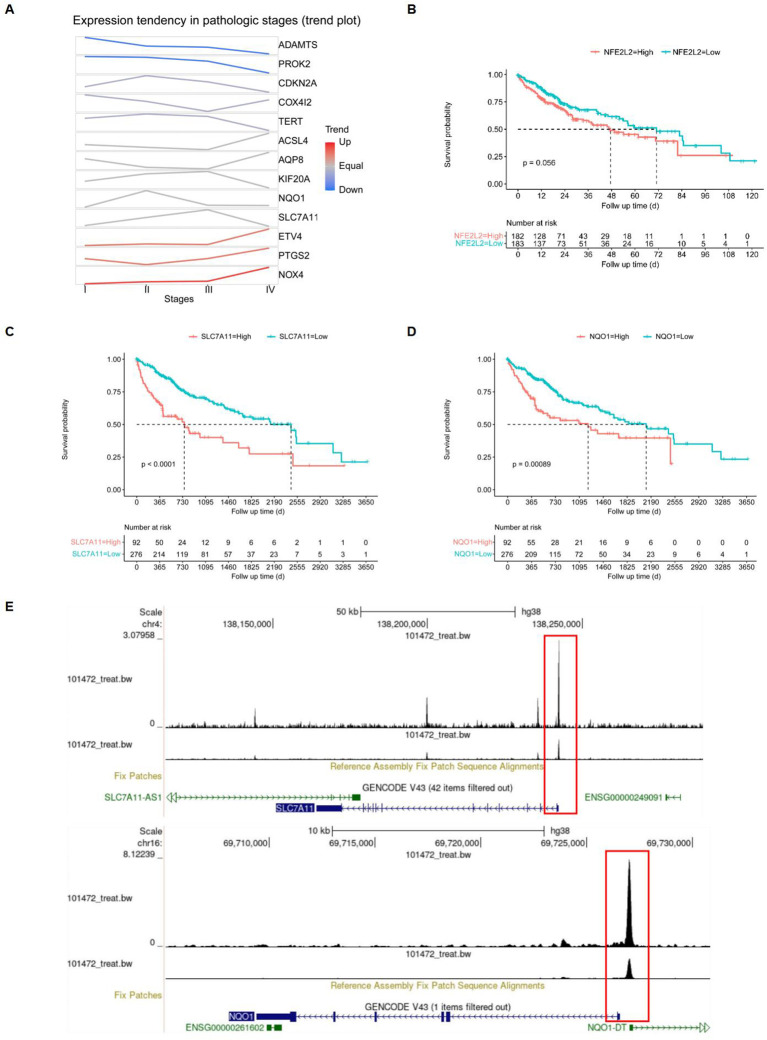
Screening of target genes. **(A)** FRGs expression tendencies in pathologic stages. **(B-D)** Kaplan-Meier overall survival (OS) curves for patients with high- and low-expressing NFE2L2, SLC7A11, and NQO1, with red indicating high expression and blue indicating low expression. **(E)** Gene transcription regulation of NFE2L2.

After identifying the 13 differentially expressed FRGs, we employed the GSCA database to analyze mRNA expression trends from the early to late stages of HCC. The trend maps exposed distinct patterns of change at different stages, with a focus on the five risk genes: ETV4, KIF20A, CDKN2A, SLC7A11, and NQO1. It was observed that the expression levels of these genes collectively increased from stage I to stage II. Between stage II and stage III, the expression of KIF20A and SLC7A11, among the five risk genes, exhibited a significant increase. Finally, from stage III to stage IV, an increase in expression was observed solely for ETV4. (**Figure 8A**). Among the five risk genes identified, SLC7A11 and NQO1 have been recognized as critical players in conferring resistance to ferroptosis (32, 33). SLC7A11 is essential for regulating GSH synthesis, which confers resistance to ferroptosis. NQO1, an enzyme, protects cells from oxidative stress, thereby reducing their susceptibility to ferroptosis. Both genes facilitate tumor growth by inhibiting ferroptosis. We categorized samples into high- and low-expression groups based on the levels of NFE2L2, SLC7A11, and NQO1, with higher expression levels being associated with poorer survival outcomes in the TCGA database, showing significant p-values for SLC7A11 (*p* < 0.0001) and NQO1 (p = 0.00089) (**Figures 8B–D**). The NFE2L2 gene encodes NRF2, a regulator of cellular antioxidant responses. Analysis of NRF2 chromatin immunoprecipitation followed by sequencing (ChIP-seq) data revealed that NRF2 binds in the vicinity of key antioxidant target gene loci, specifically NQO1 and SLC7A11, as documented in the Gene Transcription Regulation Database, indicating NRF2's role in their transcriptional regulation, which is essential for combating oxidative stress (**Figure 8E**).

### 3.7 Drug resistance analyses in high- and low-expression groups of genes that are sensitive to ferroptosis

**Figure 9:**
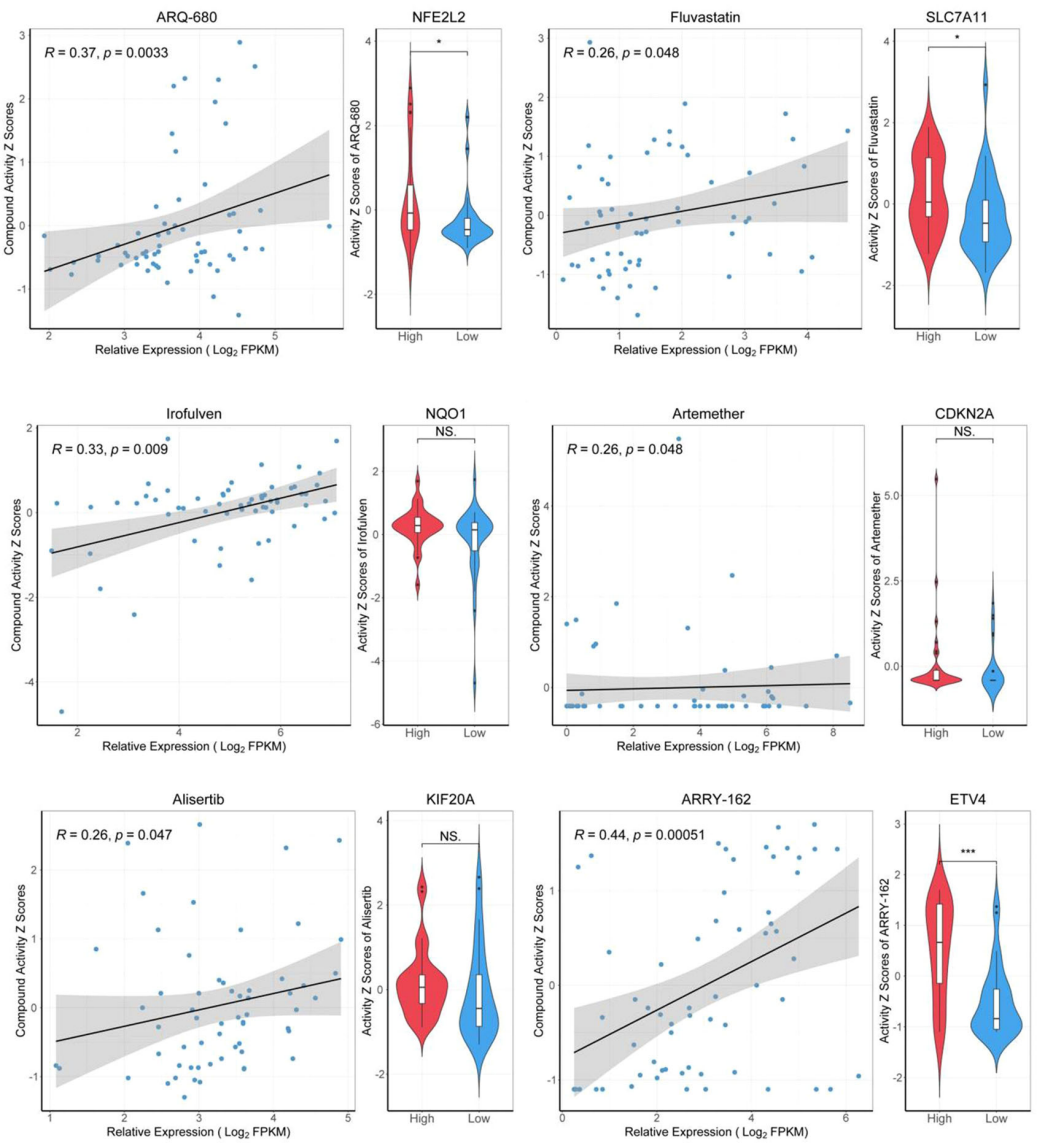
Drug sensitivity analysis of target genes. "High" representing high-expressed genes, and "Low" representing low-expressed genes. *p<0.05, ***p<0.001.

We retrieved data from the CellMiner database, which included drug activity measurements (drug-tolerant persister NCI-60-Average z score) and gene expression profiles (RNA-seq composite expression) for NFE2L2, NQO1, KIF20A, ETV4, SLC7A11, and CDKN2A in HCC cell lines. Employing the "pattern comparison" tool, we conducted comparative analyses to explore the relationship between gene expression and drug activity. We found that an improved prognosis is correlated with increased drug binding sensitivity, potentially mitigating drug resistance. These six genes, known to inhibit ferroptosis, display significant expression variation within our HCC gene dataset. As gene expression in liver cancer cells increases, so does the binding affinity between the drugs and the cancer cells. Consequently, a positive correlation between the binding potency of certain drugs and gene expression levels was established. We prioritized drugs with the strongest binding affinity, suggesting their potential for the most significant therapeutic impact (**Figure 9**).

### 3.8 Validation of the expression levels of NRF2, SLC7A11, and NQO1 in human liver and HCC tissues

**Figure 10:**
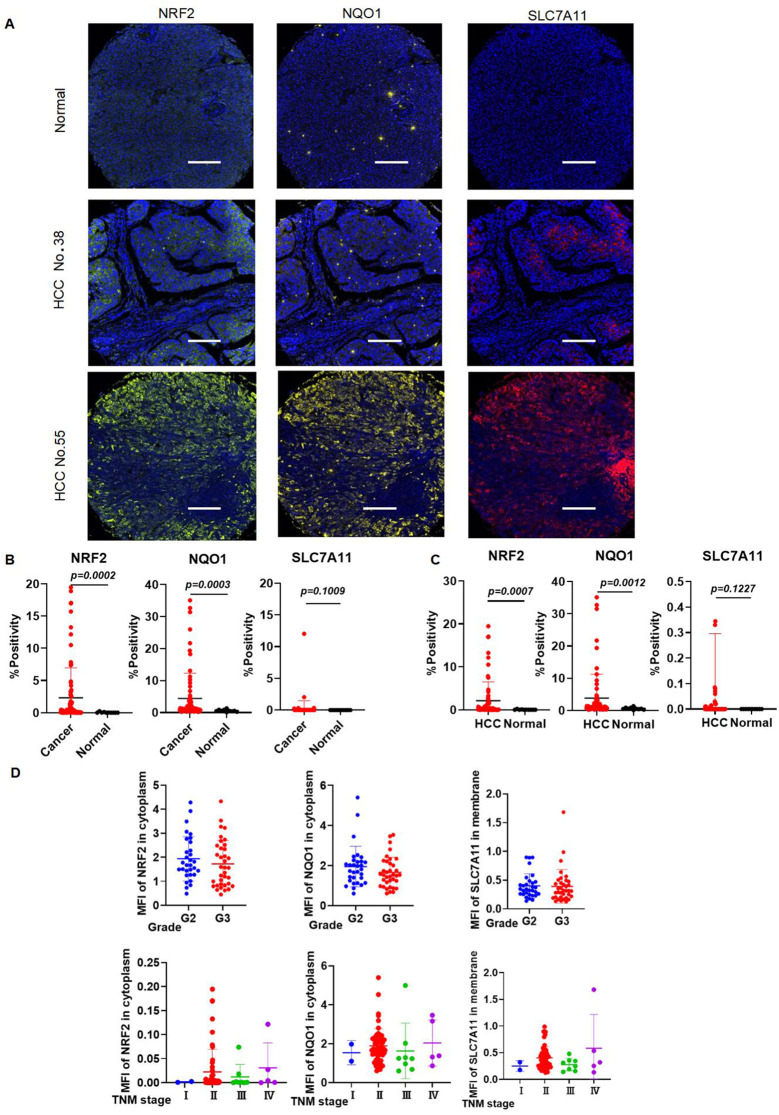
The expression levels of NRF2, SLC7A11, and NQO1 in HCC are higher in HCC than those in healthy individuals. **(A)** Representative images of mIHC staining of NRF2, SLC7A11, and NQO1. **(B)** mIHC statistical analysis of NRF2, SLC7A11, and NQO1 in all liver cancer and healthy individuals. **(C)** mIHC statistical analysis of NRF2, SLC7A11, and NQO1 in HCC and healthy individuals. **(D)** The expression of NRF2, NQO1, and SLC7A11 in different grades and TNM stages.

The general and clinical characteristics of the study participants, as determined by the high-throughput tissue microarray, were presented in **Table 1**. mIHC analysis confirmed the expression patterns of NRF2, SLC7A11, and NQO1 proteins in HCC tissues compared to normal liver tissues. Notably, NRF2, SLC7A11, and NQO1 co-localized, with an increased positive rate for SLC7A11 and NQO1 associated with a high NRF2 positive rate in patients (**Figure 10A**). The positive rates for NRF2 and NQO1 were significantly higher in liver cancer tissues than in normal liver tissues. However, in the results of the local scan analysis, SLC7A11 did not exhibit this differential expression between all liver cancer and healthy individuals. Consequently, in subsequent analyses, it is necessary to either examine the differential positive rate for each cell or increase the sample size (**Figure 10B**). The positive rates for NRF2 and NQO1 in HCC tissue microarrays were also significantly greater than those in normal liver tissue microarrays. SLC7A11 did not demonstrate a significant difference between HCC patients and healthy individuals in the local scan analysis (**Figure 10C**). We combined the mean fluorescence intensity (MFI) values from the local scan areas with clinical data to evaluate whether the expression levels of NRF2, NQO1, and SLC7A11 varied significantly across different grades and tumor-node-metastasis (TNM) stages. However, no statistical differences were observed (**Figure 10D**).

**Table 1:** General and clinical characteristics of liver cancer patients (N=96).

	Fall Group (n=86)		Control Group (n=10)	
Characteristics	Mean±SD or n (%)			
Age	50.95±9.95		35.60±10.16	
Sex				
F	15	(0.17)	7	(0.70)
M	71	(0.83)	3	(0.30)
Grage				
2	42	(0.51)	–	–
2-3	2	(0.02)	–	–
3	38	(0.46)	–	–
Stage				
I	10	(0.12)	–	–
II	62	(0.72)	–	–
III	9	(0.10)	–	–
IV	5	(0.06)	–	–

### 3.9 Correlation of NRF2 with NQO1 and SLC7A11 in human liver and HCC tissues

**Figure 11:**
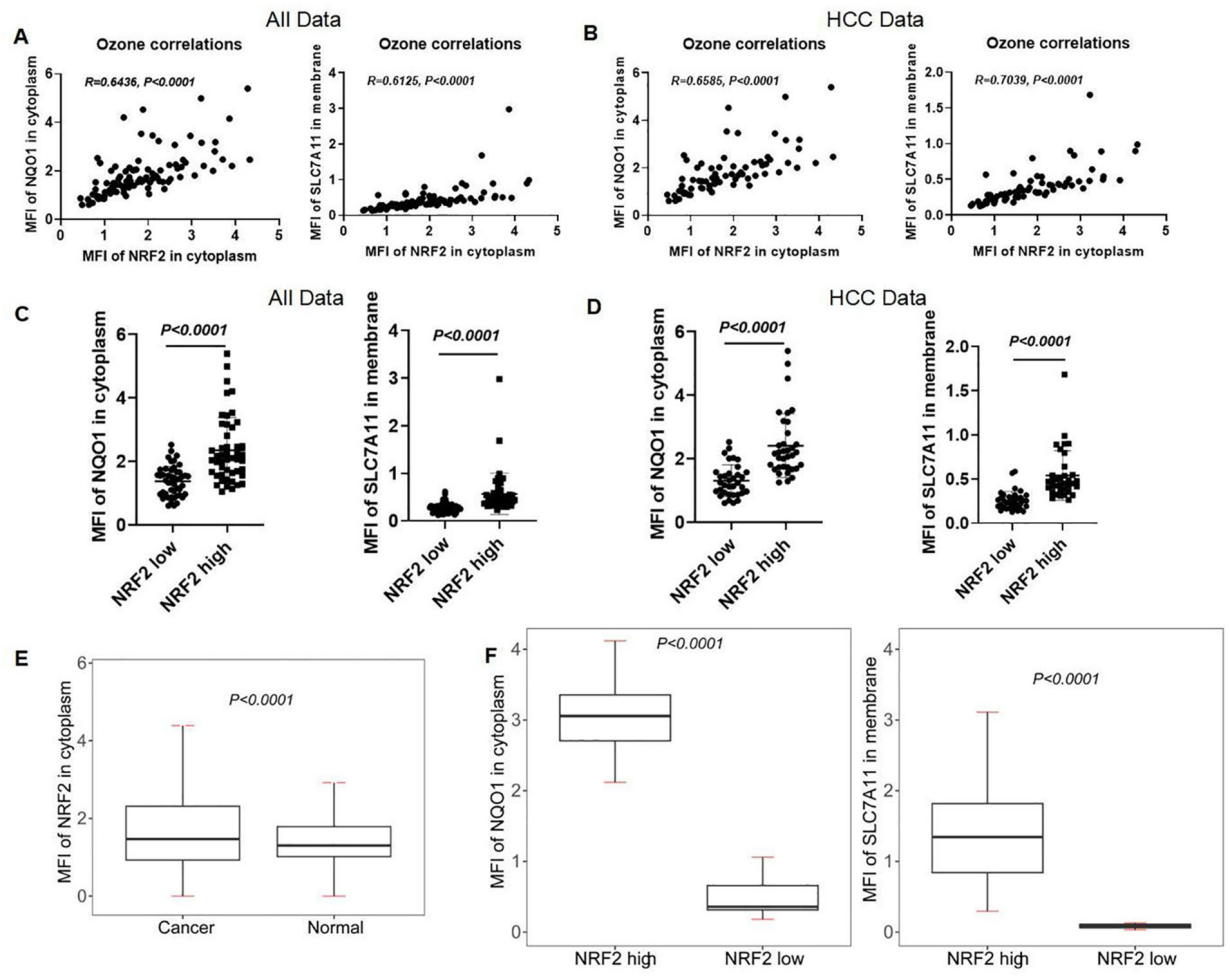
Correlation of NRF2 with NQO1 and SLC7A11 in human liver and HCC tissues. **(A, B)** The correlation analysis of NRF2, NQO1, and SLC7A11 in all subjects and HCC patients. **(C, D)** mIHC MFI statistical analysis of NQO1 and SLC7A11 in the local scan of all subjects and HCC patients with high and low expression of NRF2. **(E)** mIHC MFI statistical analysis of NRF2 in full scan between all liver cancer patients and normal people. **(F)** The different MFIs of NQO1 and SLC7A11 in HCC patients with high and low expression of NRF2.

We analyzed the correlation between NRF2 and NQO1, SLC7A11 in LIHC using data from the analysis data by the inform software after scanning. The expression levels of NRF2 showed a positive correlation with both NQO1 (r = 0.6436, *p* < 0.0001) and SLC7A11 (r = 0.6125, *p* < 0.0001) in LIHC patients (**Figure 11A**). This correlation was also observed in HCC patients, where NRF2 expression levels were positively correlated with NQO1 (r = 0.6585, *p* < 0.0001) and SLC7A11 (r = 0.7039, *p* < 0.0001) (**Figure 11B**). We categorized the MFI data of NRF2 in the cytoplasm of LIHC patients and healthy individuals into "NRF2 high" and "NRF2 low" based on the median MFI values of local scan slices. Elevated MFI of NRF2 in the cytoplasm was found to correlate with increased MFI of SLC7A11 in the membrane and NQO1 in the cytoplasm (**Figure 11C**). This pattern was consistent when analyzing HCC data alone, indicating a uniform association across patient samples (**Figure 11D**). At the single-cell level, significant differences (*p* < 0.0001) in the NRF2 expression patterns were identified between all liver cancer patients and healthy individuals (**Figure 11E**). We then classified the single-cell mean MFI data of NRF2 in the cytoplasm for both HCC patients and healthy individuals, selecting the highest MFI values from five HCC patients and the lowest from five healthy individuals. This approach confirmed that elevated MFI of NRF2 in the cytoplasm correlates with increased MFI of SLC7A11 in the membrane and NQO1 in the cytoplasm (**Figure 11F**).

### 3.10 DIC sensitizes IKE-induced ferroptosis in HCC cell lines and leads to tumor regression

**Figure 12:**
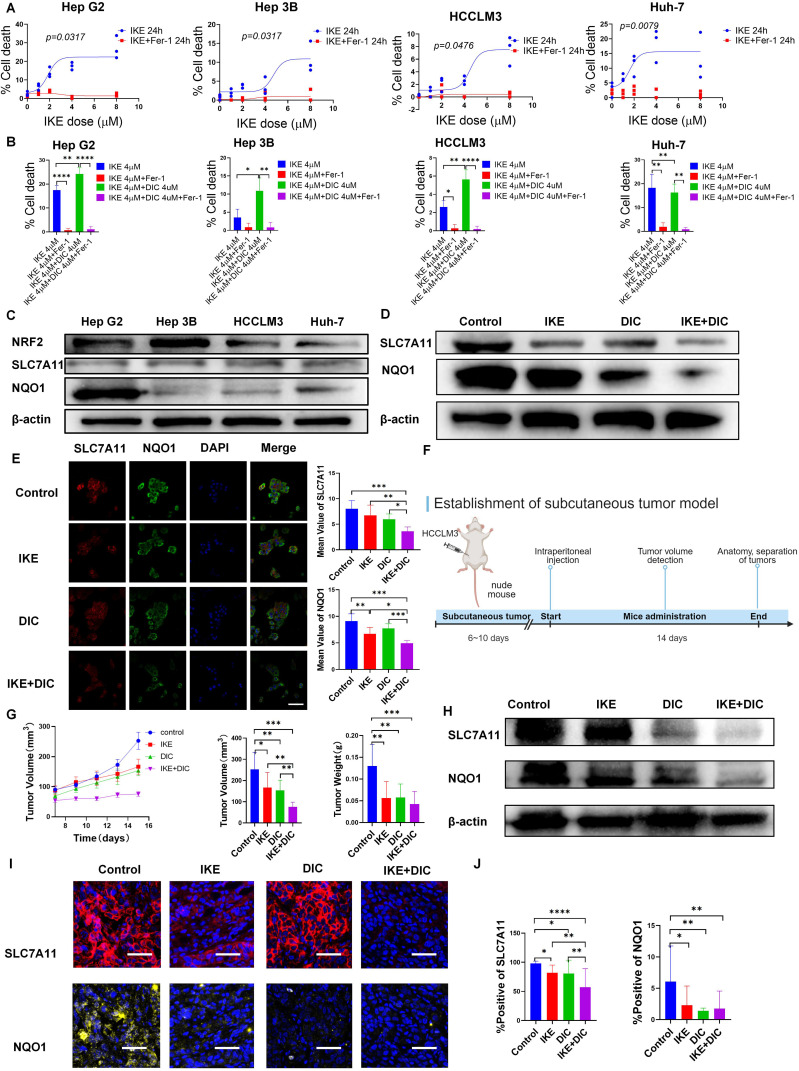
Establishment of subcutaneous tumor model. **(A)** The image shows the Hep G2, Hep 3B, HCCLM3, and Huh-7 were treated with IKE (0, 1, 2, 4, 8 μM) and IKE (0, 1, 2, 4, 8 μM) + Fer-1 (2 μM) respectively. Cell death was measured following 24-h treatment of IKE and Fer-1. **(B)** The image shows the Hep G2, Hep 3B, HCCLM3, and Huh-7 were treated with IKE (4 μM), IKE (4 μM)+Fer-1 (2 μM), IKE (4 μM)+DIC (4 μM), and IKE (4 μM)+DIC (4 μM)+ Fer-1 (2 μM) respectively. Cell death was measured following 24-h treatment of IKE, DIC, and Fer-1. n = 3 biologically independent samples per condition. **(C)** The expression of target protein in Hep G2, Hep 3B, HCCLM3, and Huh-7. **(D)** Protein expression of target molecules of HCCLM3 cells. **(E)** Immunofluorescence staining of SLC7A11 and NQO1 in HCCLM3 cells. Scale bars for others, 100 μm. **(F)** The establishment of a subcutaneous tumor model schematic diagram. **(G)** Subcutaneous tumor volume monitoring. **(H)** Protein expression of target molecules in subcutaneous tumor. **(I)** Multiple immunohistological staining of SLC7A11 and NQO1 in subcutaneous tumor. Scale bars for others, 100 μm. **(J)** Multiple immunohistological staining positive rate statistics of SLC7A11 and NQO1. *p<0.05, **p<0.01, ***p<0.001, ****p<0.0001.

Cell death induced by IKE was suppressed by the ferroptosis inhibitor Ferrostatin-1 (Fer-1). All cell lines showed sensitivity to IKE-induced ferroptosis in a dose-dependent manner, with Hep G2 cells being the most sensitive and HCCLM3 cells the least sensitive (**Figure 12A**). To investigate whether DIC can enhance IKE-induced ferroptosis, we selected HCCLM3 cells for our further research (**Figure 12B**). NRF2, NQO1, and SLC7A11 were confirmed to be expressed in HCC cell lines, including Hep G2, Hep 3B, HCCLM3, and Huh-7. (**Figure 12C**). We verified the effects of IKE and DIC on the expression of SLC7A11 and NQO1 *in vitro* using western blot and immunofluorescence experiments (**Figures 12D, E**). A subcutaneous tumor model was established using HCCLM3 cells in nude mice to investigate the impact of NQO1 and SLC7A11 on tumor growth (**Figure 12F**). The combination of DIC with IKE significantly decreased tumor growth (**Figure 12G**). The expression levels of SLC7A11 and NQO1 were significantly reduced in groups treated with IKE, DIC, or both, with the combined treatment showing a more pronounced decrease compared to single treatments (**Figures 12H–J**). These findings indicated that the combination of SLC7A11 and NQO1 inhibitors is a promising therapeutic approach for HCC.

**Figure 13:**
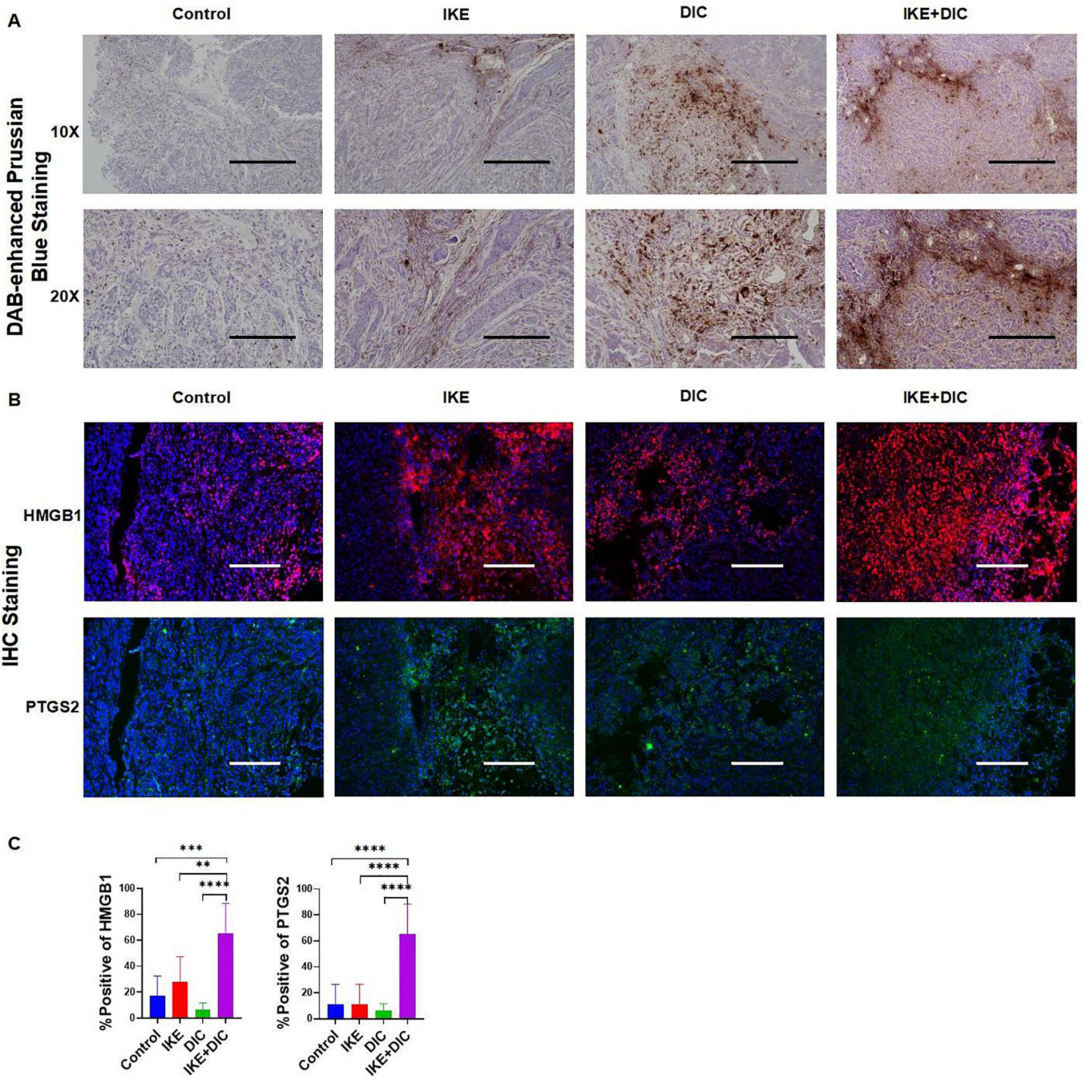
DIC enhances susceptibility to IKE-induced ferroptosis by increasing non-heme iron. **(A)** DAB-enhanced Prussian blue staining for iron in sections obtained from subcutaneous tumors. **(B)** Representative images of mIHC of HMGB1 and PTGS2 in subcutaneous tumors. **(C)** mIHC positivity statistical analysis of HMGB1 and PTGS2 in a partial scan of mice. **p<0.01, ***p<0.001, ****p<0.0001.

The combination of IKE and DIC significantly reduced tumor volume and weight. Our research confirmed that DIC inhibited the expression of NQO1 and IKE inhibited the expression of SLC7A11, both of which are known to suppress ferroptosis. Importantly, DIC sensitized HCCLM3 cells to IKE-induced ferroptosis. To investigate how DIC sensitizes tumor cells to IKE-induced ferroptosis *in vivo*, we used DAB-enhanced Prussian blue staining to identify iron deposits within cells and tissues. Our analysis revealed that the treatment groups—IKE, DIC, and the combination of IKE and DIC—increased the level of iron. The hepatic non-heme iron in sections from mice that IKE and DIC were higher than in those treated with IKE or DIC alone and control mice, as determined by DAB-enhanced Prussian blue staining (**Figure 13A**). mIHC showed that the expression levels of HMGB1 and PTGS2 were significantly elevated in tumors treated with IKE and DIC together (**Figures 13B, C**).

## 4 Discussion

Ferroptosis, an iron-dependent type of programmed cell death (PCD) marked by increased lipid peroxidation, is implicated with tumor growth and therapeutic responses across various cancers. While the precise effect of ferroptosis in tumor biology is not fully understood, evidence suggests a link between therapeutic induction of ferroptosis and mutations in cancer-related genes, particularly those involved in stress response pathways (34). It is hypothesized that IKE and DIC might induce ferroptosis and modulate other biological processes by regulating SLC7A11 and NQO1 expression, potentially explaining the observed reduction in tumor volume in our experiment.

The translation of ferroptosis-targeted therapies from the bench to bedside is crucial yet challenging. Several treatments, including radiotherapy, immunotherapy, chemotherapy agents (like altretamine), and targeted therapy agents (like sorafenib), are known for their potential to induce or sensitize cancer cells to ferroptosis (21). However, extensive clinical trials are necessary to confirm the safety and efficacy of these methods in combination with other ferroptosis-inducing treatments and to determine their ability to replicate preclinical successes in overcoming therapeutic resistance (34).

We employed three distinct analytical methods to assess differential gene expression in HCC using transcriptomic data from both the TCGA and GTEx databases. The intersection of differentially expressed genes from HCC with the FRGs in the FerrDb database identified a set of 13 differentially expressed FRGs. Following a comprehensive bioinformatics analysis, including functional enrichment, gene set enrichment, gene correlation assessments, the construction of a PPI network, and various regression analyses such as univariate Cox, Lasso, and multivariate regression, we developed a prognostic risk model. Within this model, SLC7A11 and NQO1 were identified as the key target genes for this investigation.

NRF2, a master regulator of cellular redox homeostasis and xenobiotic detoxification (35), is often associated with elevated NFE2L2/NRF2 expression, oncogenic activity of NRF2, tumor growth, metastasis, and resistance to anticancer treatments, primarily due to Kelch-like ECH-associated protein 1 (KEAP1) mutations (36–38). Clinical studies have correlated high NRF2 expression with poor prognosis in various malignancies (39). As cancer cells can develop resistance to apoptosis-inducing therapies, alternative PCD mechanisms, such as ferroptosis, warrant exploration (40). The expression of SLC7A11 and NQO1 is positively regulated by the transcriptional factor NRF2, whereas they are negatively regulated by the NRF2 suppressor gene KEAP1 (41). NRF2, a transcription factor, plays a crucial role as a major regulator of the antioxidant response. It promotes the transcription of SLC7A11 and NQO1 under conditions such as oxidative stress, as evidenced by references (42, 43). Notably, NRF2 enhances the mRNA level of xCT by binding to the antioxidant response element (ARE), which is also recognized as the electrophilic response element (EpRE), located in the proximal promoter region of the xCT gene (43). Upon receiving oxidative signals, NRF2 translocates to the nucleus, where it binds to ARE to enhance the mRNA level of NQO1 within the promoter regions of numerous phase II detoxification and antioxidant genes (44). Furthermore, NRF2 and the inflammasomes it activates are responsible for inflammasome-dependent HMGB1 release (45). HMGB1, in turn, promotes ferroptosis by modulating the Nrf2/HO-1 pathway (46). It is also worth mentioning that the inhibition of the p53/SLC7A11/GPX4 pathway, which is mediated by HMGB1, can effectively inhibit ferroptosis (47). Additionally, the inhibition of PTGS2 expression, coupled with the activation of the NRF2 signaling pathway and its downstream ferroptosis-related proteins, such as SLC7A11, can lead to a reduction in lipid peroxidation. This, in turn, alleviates ferroptosis induced by iron overload (45).

Our study seeks to confirm the regulatory role of NRF2 on SLC7A11 and NQO1 during HCC development and to evaluate the potential of DIC to enhance HCC susceptibility to IKE-induced ferroptosis. SLC7A11 and NQO1 have been identified as inhibitory genes of ferroptosis in previous studies. System Xc-, composed of SLC3A2 and SLC7A11 (xCT), facilitates cystine import and glutamate export. Elevated SLC7A11 expression in myeloma cells increases susceptibility to erastin-induced ferroptosis (48). Overexpression of SLC7A11 can promote tumor growth by inhibiting ferroptosis (49) and evade ferroptosis through post-transcriptional mechanisms. The inhibition of SLC7A11 has been shown to induce ferroptosis in tumor cells (30, 50). Activation of mTORC1 enhances ferroptosis resistance and tumor progression by up-regulating SLC7A11 (51). Decreased sensitivity to ferroptosis activators such as erastin has been observed in myocardial infarction models (52). IKE, an erastin analog, is recognized as an effective and metabolically stable inhibitor of the system Xc-, which can serve as a novel anti-tumor drug to inhibit tumor growth by inducing ferroptosis. The use of polyethylene glycol-poly (lactic-co-glycolic acid) nanoparticles (PEG-PLGA NPs) to facilitate IKE delivery has been highlighted in previous studies, demonstrating low toxicity in diffuse large B cell lymphoma (DLBCL) xenograft models (53). These findings suggest that the efficacy of IKE can be enhanced and its toxicity can be reduced through a specific drug delivery system, which is crucial for clinical application. NQO1, a cytoplasmic flavoprotein, is overexpressed in various cancers, including breast, pancreatic, hepatocellular, bladder, ovarian, thyroid, colorectal, cholangiocarcinoma, cervical, melanoma, and lung (54–58). Its overexpression is associated with larger tumor sizes, advanced stages, and poor survival rates (56, 58, 59). Triggering ferroptosis *via* NQO1 can also combat tumor drug resistance (30). DIC, as an NQO1 inhibitor and an FDA-approved drug, was identified as a potential therapeutic agent targeting core ferroptosis-related genes in polycystic ovary syndrome (60). Our study explores the potential of DIC to reduce IKE resistance and enhance HCC susceptibility to ferroptosis.

However, no literature has reported the synergistic effect of combining these two inhibitors to enhance the susceptibility of HCC cells to ferroptosis, thereby exerting an inhibitory effect on tumor growth. We hypothesize that the mechanism by which DIC enhances ferroptosis sensitivity to IKE may be related to the increase in non-heme iron levels. It is crucial to acknowledge that the subcutaneous xenograft model may not perfectly mimic the complex tumor microenvironment. Furthermore, since these models are typically established in immunodeficient mice, they may give rise to tumors that exhibit characteristics distinct from those of human cancers (61). Our subsequent studies will further verify the roles of IKE and DIC through patient-derived xenograft (PDX) models and patient-derived organoid (PDO) models. These models are expected to provide a more accurate representation of human cancers, allowing for a deeper understanding of the mechanisms and therapeutic potential of ferroptosis in HCC.

Although we have shown that DIC can reduce IKE resistance and increase HCC susceptibility to ferroptosis, it is essential to further verify the safety and efficacy of the combination of DIC and IKE in a larger cohort of clinical trials. This verification process should include evaluating drug interactions, determining maximum tolerated doses, assessing pharmacokinetic and pharmacodynamic properties, and considering possible long-term side effects.

In this study, we have successfully developed a nomogram model that is designed to calculate the Risk Score for HCC patients based on the expression levels of five genes that have been rigorously pre-screened for their relevance in HCC. Application of this model provides a comprehensive elucidation of the molecular signaling events that are pivotal in the pathogenesis of HCC, and it offers valuable insights into the realm of targeted therapy. Furthermore, it identifies potential biomarkers that could be utilized for the prediction of therapeutic efficacy. While significant strides have been made in the development of targeted therapies for HCC, there are still formidable challenges to be addressed in the implementation of therapeutic strategies that are both effective and precise. We put forward the hypothesis that the application of DIC could enhance the sensitivity of HCC cells to ferroptosis, a form of regulated cell death that has been implicated in cancer treatment. This enhancement could potentially serve as a critical reference point in the development of novel treatment approaches for HCC, offering a new avenue for improving patient outcomes.

## Data Availability

The data presented in the study are deposited in Figshare, accession link: https://figshare.com/s/93882e56b8a8f153ba87.
